# Cisplatin resistance in head and neck squamous cell carcinoma is linked to DNA damage response and cell cycle arrest transcriptomics rather than poor drug uptake

**DOI:** 10.20517/cdr.2025.107

**Published:** 2025-09-19

**Authors:** Ketaki Sandu, Rolf Warta, Uddipta Biswas, Wang Zhang, Patrick Michl, Christel Herold-Mende, Johanna Weiss, Dirk Theile

**Affiliations:** ^1^Internal Medicine IX - Department of Clinical Pharmacology and Pharmacoepidemiology, Heidelberg University, Medical Faculty Heidelberg, Heidelberg University Hospital, Heidelberg 69120, Germany.; ^2^Department of Otorhinolaryngology, Head and Neck Surgery, Heidelberg University, Medical Faculty Heidelberg, Heidelberg University Hospital, Heidelberg 69120, Germany.; ^3^Division of Experimental Neurosurgery, Department of Neurosurgery, Heidelberg University, Medical Faculty Heidelberg, Heidelberg University Hospital, Heidelberg 69120, Germany.; ^4^Internal Medicine IV, Heidelberg University, Medical Faculty Heidelberg, Heidelberg University Hospital, Heidelberg 69120, Germany.; ^#^Authors contributed equally.

**Keywords:** Cisplatin resistance, head and neck squamous cell carcinoma, drug uptake, DNA damage, recurrence-free survival

## Abstract

**Aim:** Cisplatin resistance in head and neck squamous cell carcinoma (HNSCC) is thought to involve both reduced drug uptake and altered molecular responses. However, the relative contribution of these mechanisms remains unclear.

**Methods:** Two HNSCC cell lines with differing sensitivity (HNO97 and HNO41) were analyzed using cytotoxicity assays, atomic absorption spectroscopy-based quantification of intracellular cisplatin, caspase 3/7 assays, Western blotting, polymerase chain reaction (PCR)-based transcriptomic analysis of DNA damage response and cell cycle arrest pathways, and RNA-seq data from The Cancer Genome Atlas (TCGA) to characterize the resistance phenotype.

**Results:** HNO97 (IC_50_ = 440 µM) was 7.6-fold more resistant to cisplatin than HNO41 (IC_50_ = 57.8 µM; *P* = 0.0286). After quantifying intracellular uptake (pg Pt/µg protein) and normalizing cytotoxicity to intracellular drug levels, HNO97 (IC_50_ = 778.9 pg Pt/µg protein) remained 5-fold more resistant than HNO41 (IC_50_ = 153.5 pg Pt/µg protein), indicating only a partial reduction in resistance (33% decrease, from 7.6-fold to 5-fold; *P* = 0.0286). At cisplatin concentrations yielding comparable intracellular exposure (HNO97: 440 µM; HNO41: 196 µM; both ≈ 725 pg Pt/µg protein), caspase 3/7 activation and induction of *CDKN1A*, *GADD45A*, *GADD45G*, and *PPP1R15A* were weaker in HNO97 than in HNO41. Notably, baseline expression of these genes was significantly higher in HNO97. In the TCGA cohort, multivariate analysis showed that high *FANCD2* expression was associated with unfavorable recurrence-free survival in platinum-treated patients (hazard ratio = 4.0; *P* = 0.011), but not in those who did not receive platinum chemotherapy.

**Conclusion:** Cisplatin resistance in HNSCC appears to be driven primarily by molecular mechanisms involving DNA damage response and cell cycle arrest pathways, rather than poor drug uptake.

## INTRODUCTION

Cisplatin remains a cornerstone in the treatment of head and neck squamous cell carcinoma (HNSCC), whether used as monotherapy, in combination with other agents, or alongside radiotherapy^[1-3]^. However, treatment efficacy - and consequently long-term survival - remains unsatisfactory, largely due to intrinsic or acquired resistance to cisplatin in HNSCC^[4]^. Extensive research has investigated the mechanisms underlying cisplatin resistance in HNSCC, which can generally be divided into two major categories^[5,6]^. The first involves reduced cisplatin uptake and limited intracellular accumulation^[7]^. Besides passive diffusion, cisplatin enters and exits cells via various transmembrane drug transporters. Altered expression or activity of these transporters can significantly influence cellular drug levels and thereby confer resistance^[7]^.

The second mechanism relates to altered molecular responses to cisplatin. Among the many factors implicated, the upregulation of genes and proteins involved in DNA damage response pathways is particularly important. For instance, enhanced repair of platinum-DNA adducts by the nucleotide excision repair machinery (e.g., *ERCC1*, *XPF*) or the Fanconi anemia/BRCA pathway (e.g., *FANCD2*, *BRCA1*) has been well documented and linked to cisplatin resistance in various tumor types^[6,8]^, including HNSCC^[9-14]^. Moreover, genes involved in cell cycle regulation and tumor suppression (e.g., *CDKN1A*) have also been associated with resistance in HNSCC^[15-17]^. Taken together, cisplatin resistance in HNSCC reflects a multifaceted interplay between reduced drug uptake and altered molecular responses. The relative contribution of these mechanisms, however, remains unclear and was therefore systematically evaluated and quantified in this study.

## METHODS

### Materials

Cell culture flasks and multi-well plates were obtained from Greiner (Frickenhausen, Germany). Dulbecco’s Modified Eagle Medium (DMEM) and fetal calf serum (FCS) were purchased from PAN-Biotech (Aidenbach, Germany). Phosphate-buffered saline (PBS), penicillin-streptomycin (100×), and the GeneElute Mammalian Total RNA Miniprep Kit were purchased from Sigma-Aldrich (Taufkirchen, Germany). The Pierce^TM^ BCA Protein Assay Kit and Pierce^TM^ Detergent Compatible Bradford Reagent were obtained from Thermo Scientific (Rockford, USA). Laemmli buffer was obtained from Bio-Rad (Munich, Germany). Antibodies for Western blotting were purchased from Cell Signaling Technology (Danvers, USA). Luminata Forte ECL reagent was obtained from Millipore (Darmstadt, Germany). The RT^2^ First Strand Kit and RT^2^ polymerase chain reaction (PCR) DNA Damage and Signaling Pathway Arrays were purchased from Qiagen. The Caspase-Glo 3/7 Assay was obtained from Promega (Mannheim, Germany). Cisplatin (1 mg/mL stock in physiological (0.9%) sodium chloride) was provided by the Heidelberg University Hospital pharmacy.

### Cell lines

The HNSCC cell lines HNO97 (tongue, T3N2bM0, histologic grade 4) and HNO41 (tonsil/oropharynx, T2N2bM0, histologic grade 2) were established from intraoperatively obtained samples and have been extensively characterized^[18]^. Cells were cultured in DMEM supplemented with 10% heat-inactivated FCS, 100 U/mL penicillin, and 100 μg/mL streptomycin sulfate.

### Viability assay

Cell viability was assessed using crystal violet staining, as previously described^[19]^. Cells were seeded at a density of 3 × 10^4^ cells per well in 96-well microtiter plates and allowed to attach for 24 h at 37 °C. The first column of wells contained no cells and was used to measure background absorbance. The second column contained untreated cells, representing 100% viability. All other columns were exposed to varying concentrations of cisplatin (1 to 1,000 µM). Each concentration was tested in octuplicate, and each assay was repeated four times for both cell lines. After a 2.5-h incubation, the medium in all wells was replaced with drug-free medium, and cells were further incubated for 24 h at 37 °C. The next day, cells were washed once with PBS and stained with 50 µL of crystal violet solution (0.5% w/v in 20% methanol). Wells were then washed three times with tap water to remove unbound dye and dried at 37 °C. Crystal violet was solubilized by adding 200 µL of pure methanol to each well, followed by shaking for 10 min at room temperature. Absorbance was measured at 555 nm using a SpectraMax iD3 Multi-Mode Microplate Reader (Molecular Devices). For quantification, the mean absorbance of cell-free wells was subtracted from each sample. Cell viability was calculated as the ratio of absorbance in drug-treated wells to that in untreated wells (100% viability). Sigmoid concentration-response curves and IC_50_ values were generated using GraphPad Prism version 9 (GraphPad Software Inc., La Jolla, USA). Fold-change in cisplatin resistance was determined by normalizing replicate IC_50_ values of HNO97 to the mean IC_50_ of HNO41.

### Cellular platinum quantification in cisplatin-treated cells

Cells were seeded at a density of 1 × 10^6^ cells per well in 6-well plates (six replicates) and preincubated at 37 °C for 24 h. The cells were then treated with the same cisplatin concentrations used in the proliferation assay. After a 2.5 h exposure, the drug-containing medium was discarded, and the cells were washed once with PBS. Cells were harvested by scraping in 150 μL of 0.4% HNO_3_ and lysed by sonification for 30 min. The lysates were centrifuged at 17,000 × *g* for 3 min, and the supernatants were collected for platinum quantification using a PinAAcle 900Z graphite furnace atomic absorption spectrometer (PerkinElmer). Platinum detection was based on the background-corrected absorption peak at 265.94 nm following injection of a 20 µL sample into the furnace. The following atomic absorption spectroscopy program was used: 30 s at 110 °C, 30 s at 130 °C, 20 s at 1,300 °C, 5 s at 2,200 °C, and 3 s at 2,450 °C. A weighted linear calibration curve was constructed, using five calibration standards spanning the expected concentration range. Three quality control samples (low, middle, and high concentrations) were included. All calibration standards, quality controls, and experimental samples were measured in duplicate and were required to meet US Food and Drug Administration (FDA) criteria for accuracy and precision to be included in the final analysis^[20]^.

Platinum concentrations were normalized to the protein content of each sample, determined with a commercial bicinchoninic acid (BCA) assay kit. Briefly, a calibration curve was prepared using nine concentrations of bovine serum albumin (BSA, 0-2,000 µg/mL), dissolved in the same cell lysis buffer (0.4% HNO_3_). Wells of a 96-well plate were loaded with 8 µL of either BSA standards or supernatants, along with 64 µL of the working solution (a mixture of reagents A and B provided in the kit). After 30 min of light-protected incubation at 37 °C, absorbance was measured at 562 nm using a SpectraMax plate reader (Molecular Devices, München, Germany).

### Caspase 3/7 assay

Caspase 3/7 activity in response to similar intracellular platinum concentrations was assessed in HNO97 and HNO41 cells using the Caspase-Glo 3/7 Assay (Promega, Mannheim, Germany). Cells were seeded at 3 × 10^4^ cells per well in white-bottom 96-well plates and incubated for 24 h at 37 °C. Based on the known cisplatin uptake characteristics of HNO97 and HNO41, cells were treated with extracellular cisplatin concentrations previously validated to yield similar total intracellular platinum levels. After 2.5 h of exposure, the medium was replaced with 50 µL of fresh medium. Caspase 3/7 activity was then measured at the following time points: 0 h (immediately after the 2.5-h treatment) and 4, 8, 12, 18, and 24 h post-treatment. Prior to reagent addition, both the plates and the detection reagent were equilibrated to room temperature. Then, 50 µL of reagent was added to each well, the plates were shaken for 30 s on a rotary shaker, and subsequently incubated for 30 min at room temperature. Luminescence was measured using a SpectraMax iD3 Multi-Mode Microplate Reader (Molecular Devices, Wokingham, UK), and caspase 3/7 activity was normalized to the mean of untreated control wells at the corresponding time point.

### Western blotting

Cleavage of Caspase 3 and PARP was assessed 4 h after the 2.5-h cisplatin treatment. Cells were seeded at 2 × 10^6^ cells per T25 flask (four independent experiments) and incubated overnight at 37 °C. They were then treated for 2.5 h with cisplatin concentrations validated to produce comparable total intracellular platinum levels. Following drug exposure, cells were transferred to drug-free medium for 4 h. The medium was then removed, cells were washed with PBS and harvested by trypsinization. The resulting pellets were washed again with PBS and subjected to western blot analysis: Cell pellets were lysed in hot SDS lysis buffer containing 10 mM Tris-HCl (pH 8.0), 1% (m/v) Sodium Dodecyl Sulfate (SDS), 1× protease inhibitor cocktail, and 1× PhosSTOP phosphatase inhibitor. Protein concentration was measured using the Pierce Detergent Compatible Bradford Reagent (#23246, Thermo Fischer Scientific). Lysates were mixed with Laemmli buffer (#1610747, Bio-Rad, Munich, Germany), heated, and 20 µg of protein per sample was separated on a 12% SDS-PAGE gel and transferred to PVDF membranes. Membranes were blocked in 5% (w/v) milk/PBS-T and incubated overnight at 4 °C with the following primary antibodies (Cell Signaling Technology): cleaved caspase 3 (#9661), PARP (#9542), and GAPDH (#2118). After incubation with the secondary antibody (Cell Signaling Technology, #70745), proteins were visualized using Luminata Forte ECL (Millipore, Darmstadt, Germany).

### PCR array-based transcriptomic analysis

Based on the caspase assay results, the optimal time point for gene expression analysis was determined to be 4 h after the 2.5-h cisplatin treatment phase. Cells were seeded at 2 × 10^6^ per T25 flask (three independent experiments) and incubated overnight at 37 °C. They were then treated again for 2.5 h with cisplatin concentrations previously validated to yield comparable total intracellular platinum levels. Following drug exposure, cells were transferred to drug-free medium for 4 h. The medium was then removed; cells were washed with PBS and harvested via trypsinization. RNA was isolated from the resulting cell pellets using the GenElute^TM^ Mammalian Total RNA Kit (Sigma, Taufkirchen, Germany) according to the manufacturer’s protocol. cDNA synthesis was performed using the RT^2^ First Strand Kit (Qiagen, Hilden, Germany). The resulting cDNA was analyzed on a LightCycler 480 (Roche, Mannheim, Germany) using the RT^2^ PCR DNA Damage and Signaling Pathways Array. The RT^2^ Profiler PCR Arrays are designed to analyze panels of genes related to specific disease states or biological pathways. Each array contains primer pairs for 84 target genes and five housekeeping genes (ß-actin, ß2-microglobulin, glyceraldehyde-3-phosphate dehydrogenase, hypoxanthine phosphoribosyltransferase 1, and large ribosomal protein). In addition, one well contains a genomic DNA control, three contain reverse-transcription controls, and three contain positive PCR controls. Data analysis was performed using Qiagen’s online PCR data analysis tool, which applies the widely used ΔΔCt method^[21]^ with a combination of housekeeping genes. *P* values were calculated using Student’s *t*-test on replicate 2^-ΔΔCt^ values for each gene in the test groups compared with the control group.

### TCGA data evaluation and survival analysis

Data were obtained from The Cancer Genome Atlas (TCGA) Head and Neck Squamous Cell Carcinoma (HNSC) dataset, accessed through the Broad Institute’s Firehose repository. Clinical data, including survival information (recurrence-free survival time and status) and drug treatment details, were extracted and curated. Patients were filtered to include only those with complete survival data and corresponding RNA-Seq profiles. RNA-Seq count data were normalized using the voom function from the limma R package. For each gene/signature of interest, normalized expression values were dichotomized at the cohort median to define “high” and “low” expression groups. Recurrence-free survival between these groups was compared using Kaplan–Meier estimation and the log-rank test. Resulting *P*-values were adjusted for multiple testing using the Benjamini–Hochberg false discovery rate procedure. Genes exhibiting at least a suggestive association in univariate analyses (*P* < 0.10) were further assessed using multivariable Cox proportional hazards models to adjust for potential clinical confounders. Survival analyses were conducted using the survival R package. Statistical significance was determined using the log-rank test for univariate analysis and the Cox proportional hazards regression model for multivariable analysis.

### Statistical analysis

Cytotoxic IC_50_ values were compared using the non-parametric Mann-Whitney test. Intracellular platinum concentrations following drug treatments were also compared using the Mann-Whitney test. Differences in caspase 3/7 activity at specific time points were evaluated by Student’s *t*-test. All statistical analyses and figure generation were performed using GraphPad Prism version 9.0.0. (Boston, USA).

## RESULTS

### Cytotoxic pharmacodynamics of cisplatin

**Figure 1:**
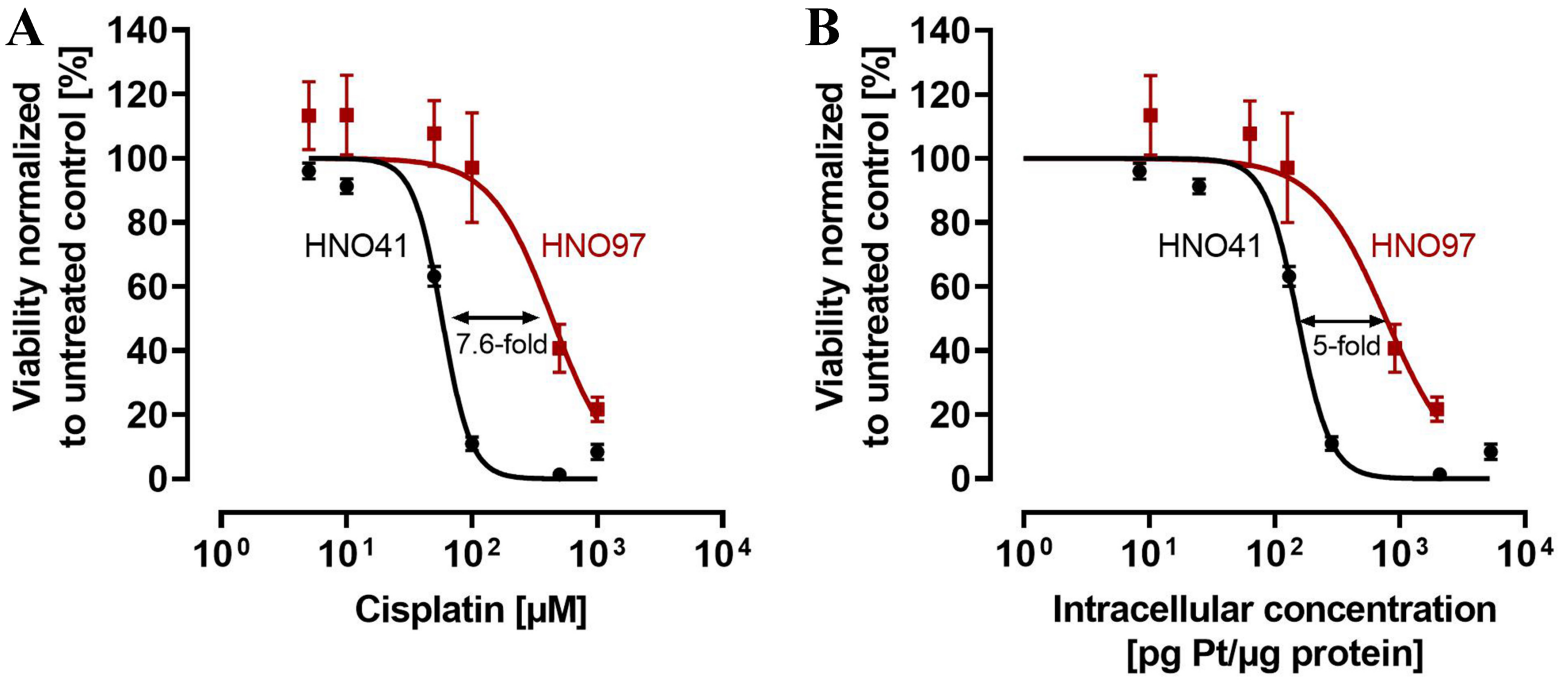
Cytotoxic pharmacodynamics of cisplatin in HNO41 (black) and HNO97 (dark red). (A) Cytotoxic effects of cisplatin as a function of nominal concentrations in the culture medium; (B) Cytotoxic effects of cisplatin normalized to total intracellular platinum concentrations. Data are presented as mean ± SD from four independent biological replicates, each with eight technical replicates. SD: Standard deviation.

When calculated based on nominal cisplatin concentrations in the culture medium, the IC_50_ for HNO41 cells was 57.8 ± 1.44 µM. In contrast, HNO97 cells were 7.6-fold (± 0.94) more resistant (IC_50_ 440 ± 54.3 µM; *P* = 0.0286; Figure 1A). However, when cytotoxic effects were normalized to total intracellular platinum concentrations, HNO97 cells (IC_50_ 778.9 ± 115.9 pg Pt/µg protein) were only 5-fold (± 0.75) more resistant than HNO41 cells (IC_50_ 153.5 ± 4.35 pg Pt/µg protein; *P* = 0.0286), corresponding to a 33% ± 0.1% (*P* = 0.0286) decrease in the difference in cisplatin resistance [Figure 1B].

### Caspase 3/7 kinetics in cells exposed to comparable total intracellular platinum concentrations

**Figure 2:**
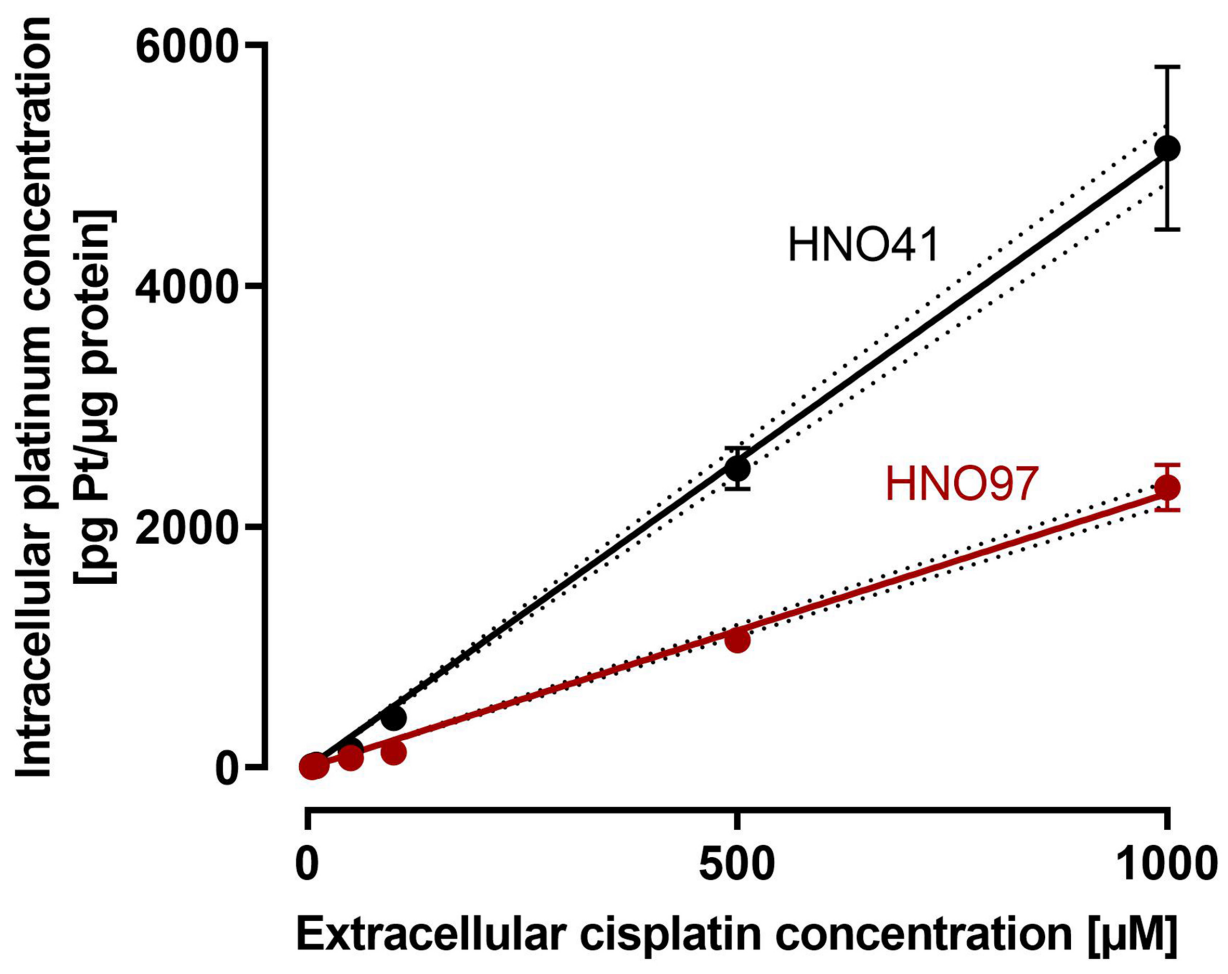
Cisplatin uptake characteristics in HNO41 (black) and HNO97 (dark red). Cells were exposed to cisplatin for 2.5 h, and total intracellular platinum concentrations were quantified. Data represent mean ± SD of four independent biological replicates. Linear regression lines with 95%CI margins (dotted lines) are depicted. SD: Standard deviation.

**Figure 3:**
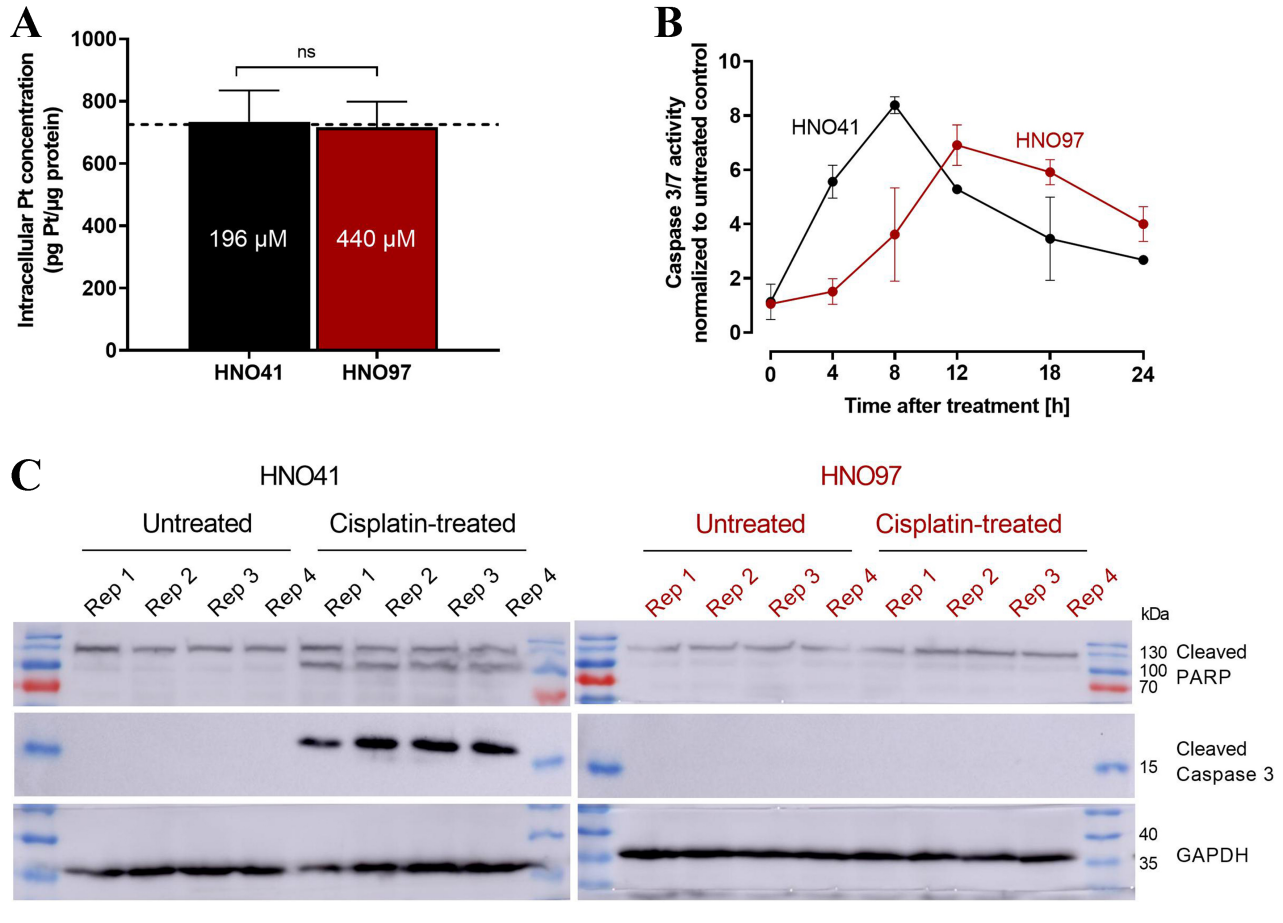
(A) Validation of similar total intracellular platinum concentrations (725 pg Pt/µg protein; dotted line) after exposure of HNO41 (black) to 196 µM cisplatin and HNO97 (dark red) to 440 µM cisplatin. Data represent mean ± SD of four independent biological replicates. Statistical significance was evaluated by the non-parametric Mann-Whitney test; ns: not significant; (B) Caspase 3/7 activity in HNO41 (black) and HNO97 (dark red) over time after 2.5 h of cisplatin treatment (HNO41: 196 µM; HNO97: 440 µM), yielding comparable intracellular platinum concentrations. Data represent mean ± SD of four independent biological replicates; (C) Western blot analysis of cleaved caspase 3 and cleaved PARP in HNO41 (black, left) and HNO97 (dark red, right), 4 h after 2.5 h of cisplatin treatment (HNO41: 196 µM; HNO97: 440 µM) or in untreated controls (four independent biological replicates each). Twenty µg of protein lysates were resolved on a 12% gel and transferred to a PVDF membrane by wet transfer. Membranes were probed with antibodies against cleaved caspase 3 and PARP. GAPDH was used as a loading control. SD: Standard deviation.

By comparing the nominal cisplatin concentrations in the culture medium with the resulting total intracellular platinum concentrations in the two cell lines, uptake characteristics could be described using linear regression. Overall, HNO97 showed reduced drug uptake compared with HNO41, as indicated by the significantly different slopes of the regression lines (HNO97 slope: 2.27 ± 0.04; HNO41 slope: 5.1 ± 0.11) [Figure 2]. Based on these linear uptake characteristics, cisplatin concentrations of 196 µM (HNO41) and 439.5 µM (HNO97) were selected to achieve similar total intracellular platinum levels (725 ± 54.3 pg Pt/µg protein; *P* > 0.9999; Figure 3A). At equivalent intracellular drug exposure, caspase 3/7 activity was monitored over time. In HNO41 cells, caspase 3/7 activity was already markedly elevated 4 h after treatment (5.5-fold compared with control), whereas no increase was observed in HNO97 at the same time point (*P* = 0.0008). Caspase 3/7 activity peaked at 8 h in HNO41 (8-fold compared with control) but was delayed until 12 h in HNO97 (7-fold compared with control) [Figure 3B]. To confirm the reduced pro-apoptotic and DNA damage responses in HNO97 cells 4 h after the 2.5-h cisplatin treatment, western blot analysis was performed. Cleaved caspase 3 (an indicator of caspase 3 activation) and cleaved PARP (a downstream target of caspase 3) were assessed. Both cleaved caspase 3 (~17 kDa) and cleaved PARP (~100 kDa) were readily detectable in cisplatin-treated HNO41 cells but absent in cisplatin-treated HNO97 cells [Figure 3C]. Notably, total PARP levels were unchanged across different cells and treatment conditions. GAPDH served as the loading control and confirmed consistent protein loading.

### Drug treatment-related changes in gene expression

**Figure 4:**
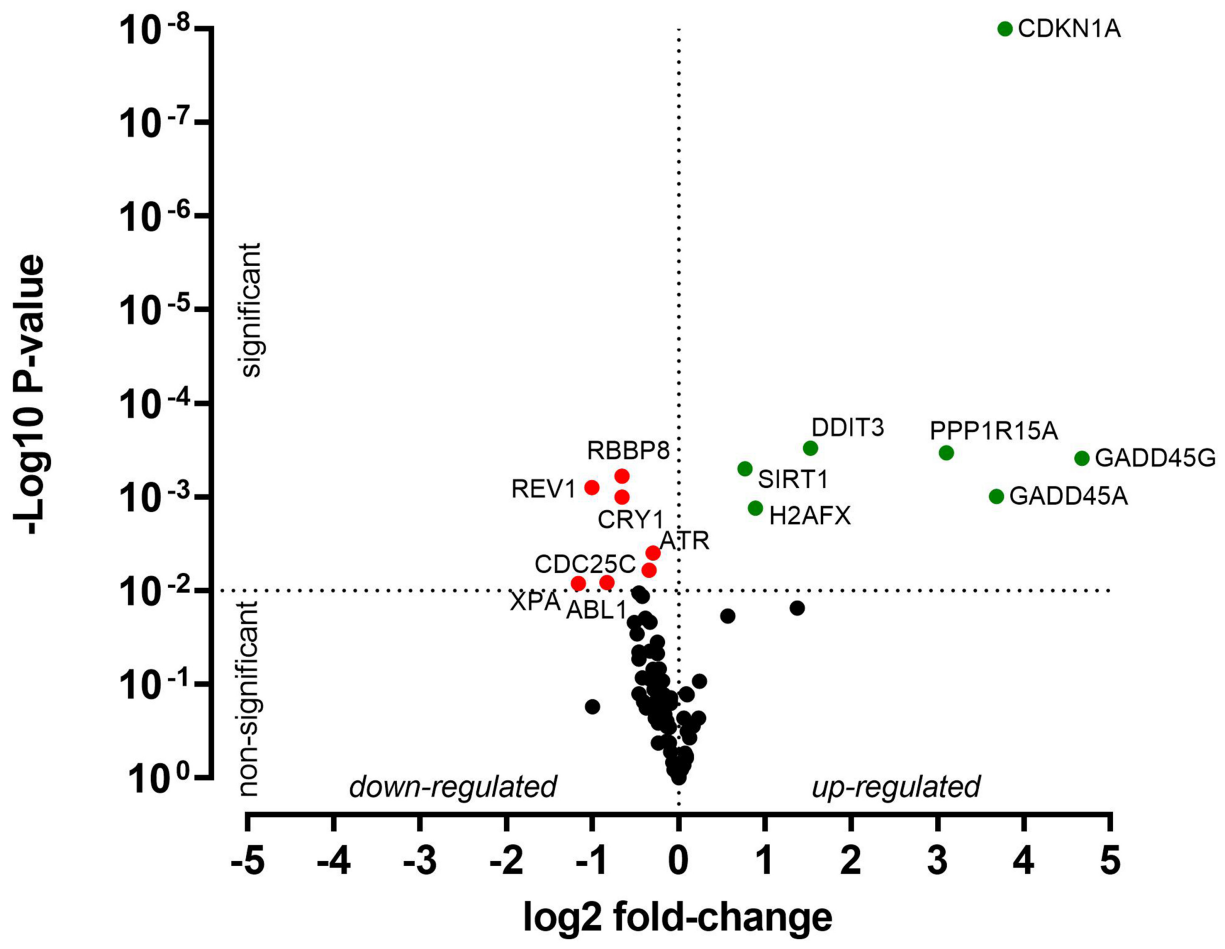
Cisplatin-induced gene expression changes in HNO41 cells. Cells (three independent biological replicates) were exposed to 196 µM cisplatin for 2.5 h, transferred to drug-free medium for 4 h, and then analyzed by PCR. Significantly induced genes are highlighted in green, significantly suppressed genes in red, and non-significantly altered genes (*P* > 0.01) in black. PCR: Polymerase chain reaction.

**Figure 5:**
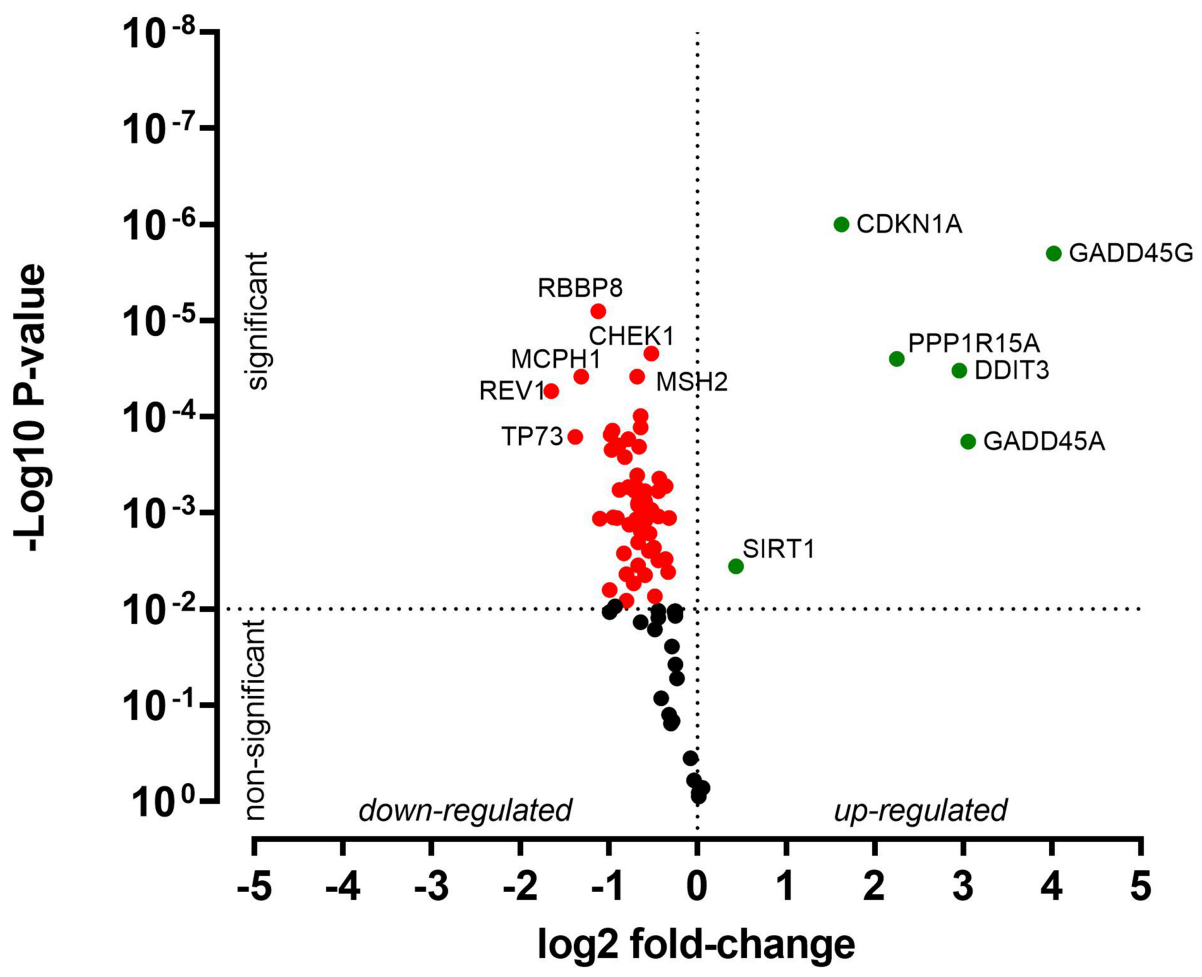
Cisplatin-induced gene expression changes in HNO97 cells. Cells (three independent biological replicates) were exposed to 440 µM cisplatin for 2.5 h, transferred to drug-free medium for 4 h, and then analyzed by PCR. Significantly induced genes are highlighted in green, significantly suppressed genes in red, and non-significantly altered genes (*P* > 0.01) in black. PCR: Polymerase chain reaction.

HNO41 and HNO97 cells were treated with cisplatin, resulting in comparable total intracellular platinum concentrations. After drug exposure, the cells were placed in drug-free medium for 4 h and then subjected to PCR array-based transcriptomic analysis targeting key DNA damage and cell cycle arrest genes. In HNO41 cells, numerous genes showed substantial changes in expression [Figure 4 and Supplementary Table 1]. For instance, *GADD45G* was induced 25-fold (2^4.67^), *CDKN1A* nearly 14-fold (2^3.78^), *GADD45A* nearly 13-fold (2^3.68^), and *PPP1R15A* 8.6-fold (2^3.1^). By contrast, HNO97 cells exhibited weaker induction of these genes [Figure 5 and Supplementary Table 2]: *GADD45G* increased only 16-fold (2^4^), *CDKN1A* 3-fold (2^1.6^), *GADD45A* 8-fold (2^3^), and *PPP1R15A* 4.75-fold (2^2.2^). In both cell lines, several genes were suppressed by approximately 50% [Figures 4 and 5, Supplementary Tables 1 and 2].

### Baseline expression levels

**Figure 6:**
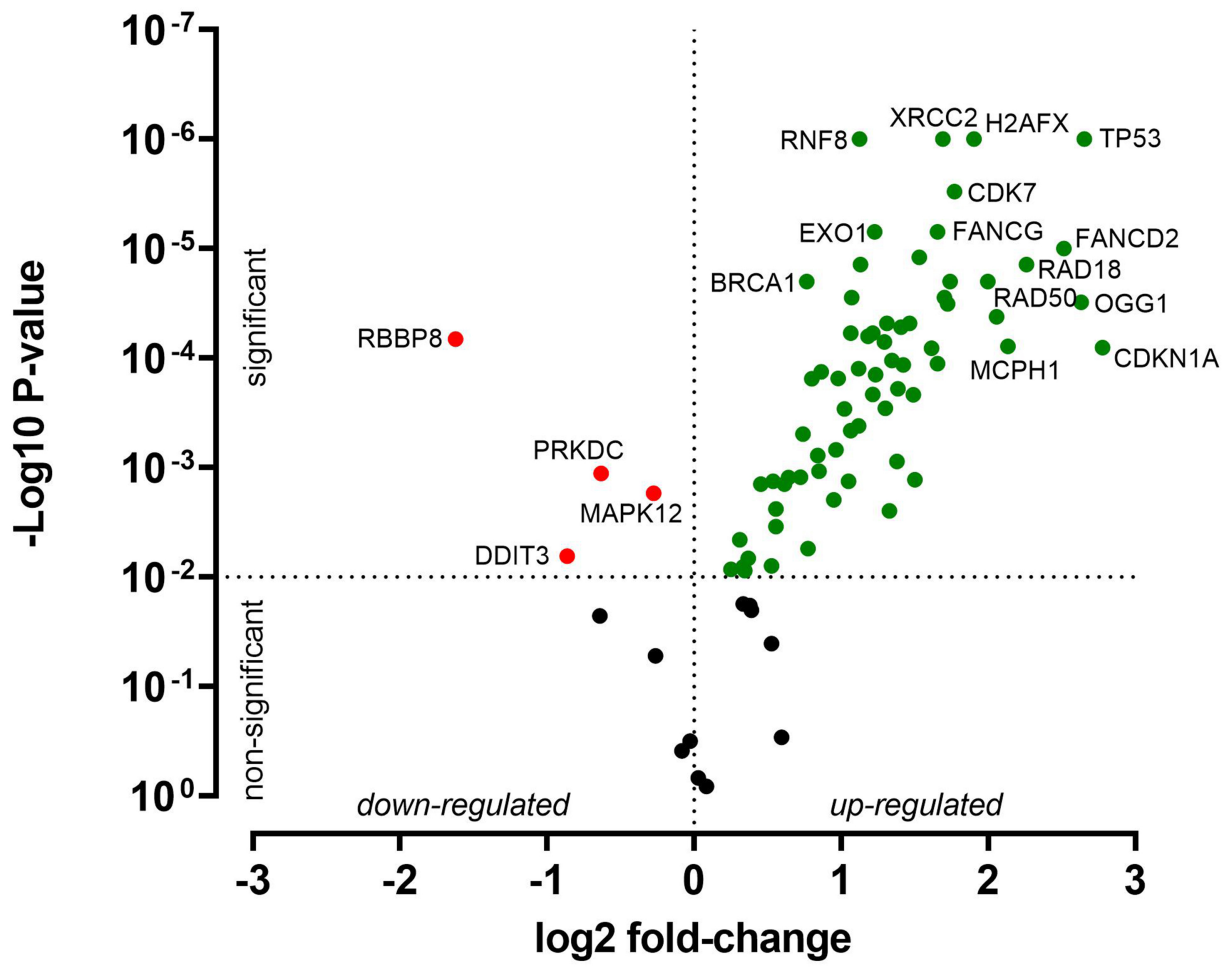
Relative baseline gene expression differences between HNO97 cells (three independent biological replicates) and HNO41 cells (three independent biological replicates). Genes significantly upregulated in HNO97 compared with HNO41 are highlighted in green, significantly downregulated genes are highlighted in red, and genes with no significant difference (*P* > 0.01) are shown in black.

**Figure 7:**
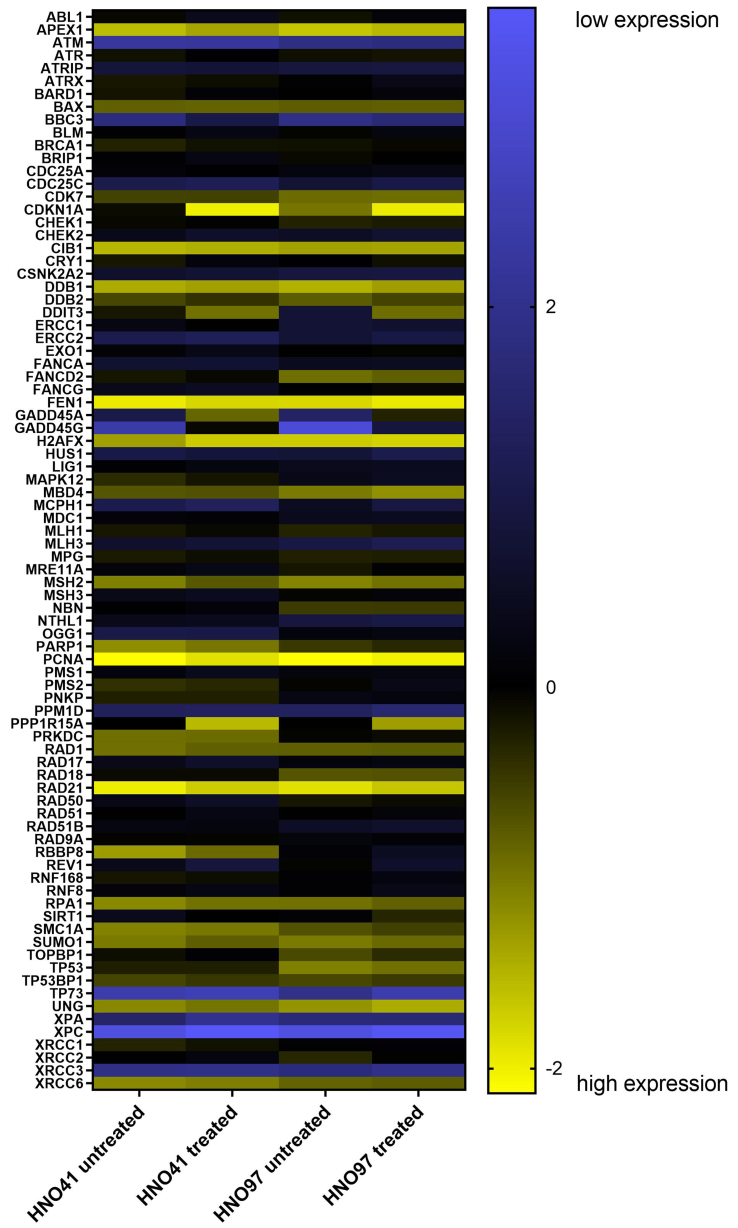
Heat map visualization of mRNA expression levels in untreated and treated HNO41 and HNO97 cells. Mean expression values from three independent replicates were Z-transformed and color-coded, with yellow indicating high expression and blue indicating low expression.

Besides treatment-related changes, baseline expression levels were also evaluated. Compared with HNO41, HNO97 cells exhibited higher mRNA expression of several genes related to DNA damage response and cell cycle arrest. For instance, *CDKN1A* expression was 7-fold higher (2^2.77^), *TP53* and *OGG1* were each 6-fold higher (2^2.6^), and *FANCD2* was 5.7-fold higher (2^2.5^) [Figure 6 and Supplementary Table 3]. The complete expression profiles of both cell lines, under baseline and drug-treated conditions, are further presented in a heat map [Figure 7].

### Clinical significance of identified candidate genes

**Figure 8:**
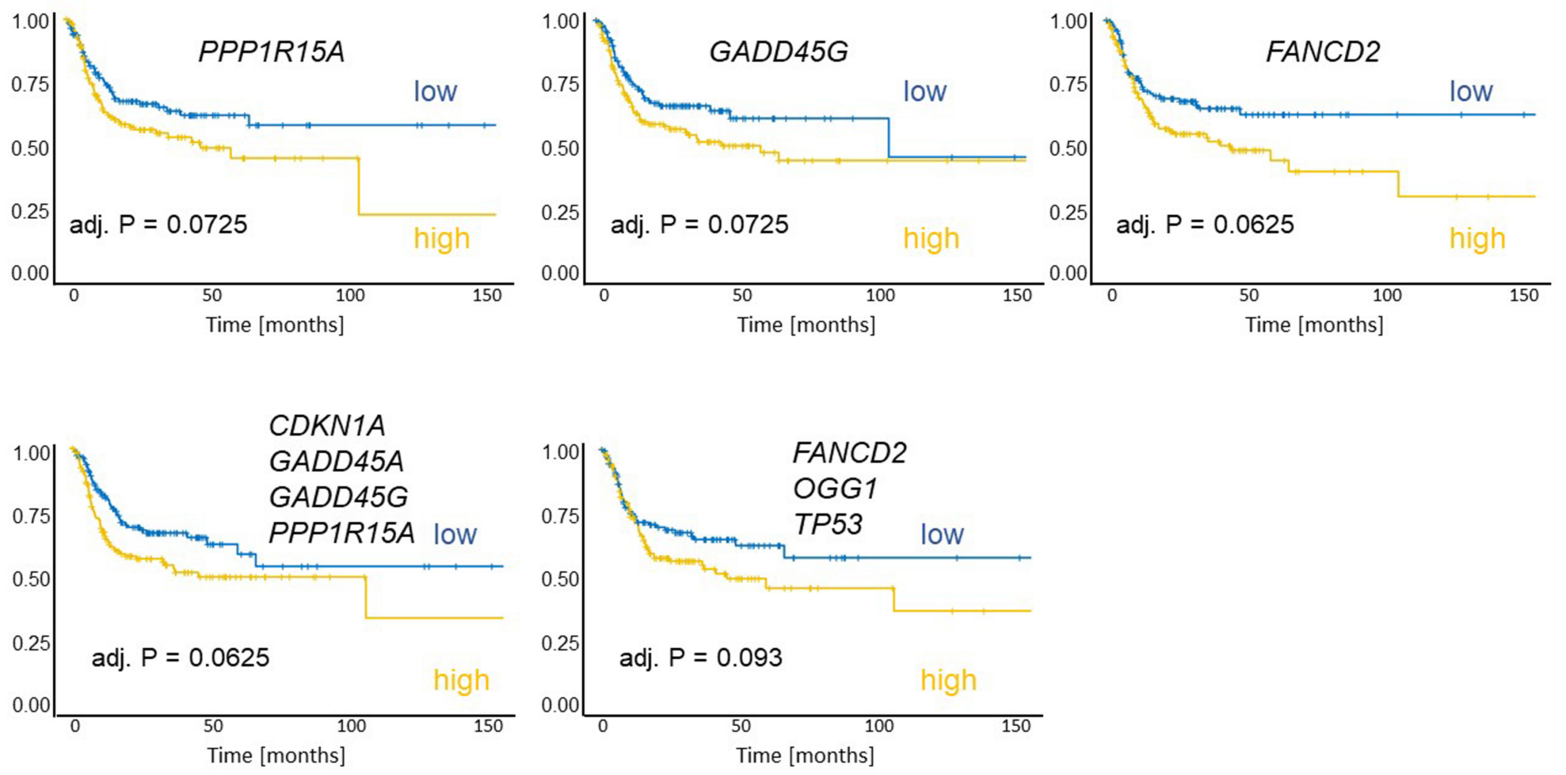
Kaplan-Meier plots of recurrence-free survival were generated for 292 patients. Patients were grouped according to the expression of individual genes (*PP1R15A*, *GADD45G*, *FANCD2*), the *in vitro* treatment-induced signature (mean of: *CDKN1A*, *GADD45A*, *GADD45G*, *PP1R15A*), and the baseline-high signature (mean of: *FANCD2*, *OGG1*, *TP53*). High expression of these genes or signatures was associated with worse outcomes. Each group comprised 146 patients.

**Figure 9:**
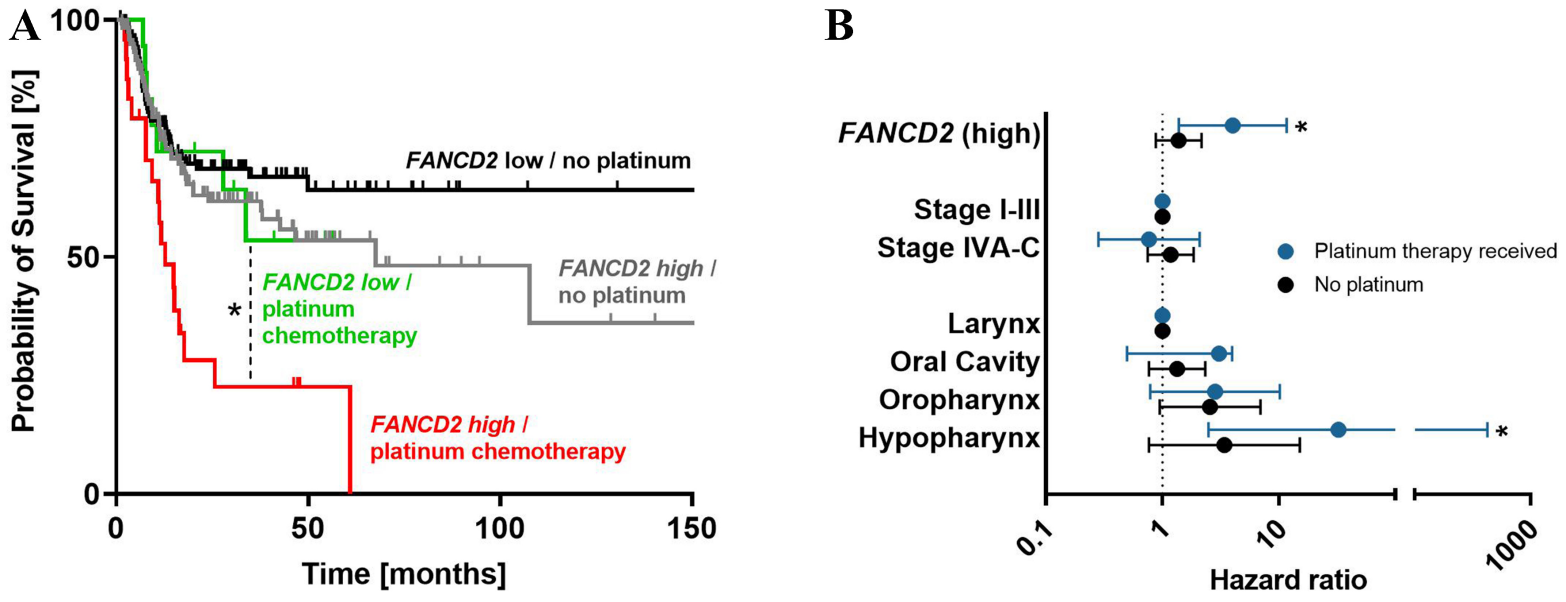
(A) Kaplan-Meier plots of recurrence-free survival stratified by *FANCD2* expression and adjuvant platinum-based chemotherapy indicate that high *FANCD2* expression predicts poor survival in patients treated with platinum-based chemotherapy; (B) This adverse effect remained significant after adjustment for clinical stage and tumor localization in multivariate Cox proportional hazard regression. ^*^P < 0.05.

To evaluate the clinical relevance of the identified genes for the survival of HNSCC patients, a TCGA-based analysis was performed. The dataset comprised 442 human papillomavirus-negative HNSCC patients (127 females, 315 males; mean age at diagnosis: 61.5 years) with tumors located in the hypopharynx (*n* = 6), larynx (*n* = 112), oral cavity (*n* = 292), or oropharynx (*n* = 32). The documented median recurrence-free survival was 17.6 months. Normalized gene expression values were dichotomized at the cohort median to define “high” and “low” expression groups. High expression of *GADD45G* (adj. *P* = 0.0725), *PPP1R15A* (adj. *P* = 0.0725), or *FANCD2* (adj. *P* = 0.0625) was associated with shorter recurrence-free survival [Figure 8]. In addition, the combined mean expression of genes strongly induced in the cisplatin-resistant HNO97 cell line (*CDKN1A*, *GADD45A*, *GADD45G*, and *PPP1R15A*) and genes highly expressed at baseline in HNO97 (*FANCD2*, *OGG1*, *TP53*) was also associated with poor recurrence-free survival (adj. *P* = 0.0625, adj. *P* = 0.093) [Figure 8]. In multivariate survival analysis, *FANCD2* remained significantly associated with poor recurrence-free survival [Table 1]. Importantly, in the subgroup of patients treated with platinum-based chemotherapy, high *FANCD2* expression (*P* = 0.011) was again associated with unfavorable recurrence-free survival [Table 2], whereas its impact was minor among patients who did not receive platinum-based chemotherapy [Table 2 and Figure 9A]. This association was further strengthened after adjusting for clinical stage and tumor site, with a significant hazard ratio of 4.0 compared to 1.4 [Figure 9B].

**Table 1:** Multivariate survival analysis of recurrence-free survival using Cox proportional-hazard regression

**Factor**		***P* value**	**Hazard ratio**	**Lower 95%CI**	**Upper 95%CI**
*FANCD2*	Low	-	-	-	-
	High	0.021^*^	1.609	1.074	2.411
Location	Larynx				
	Oral Cavity	0.178	1.398	0.859	2.277
	Oropharynx	0.011^*^	2.638	1.254	5.552
	Hypopharynx	0.013^*^	4.725	1.384	16.139
Clinical stage	I-III	-	-	-	-
	IVA-C	0.286	1.245	0.833	1.861
*In vitro* treatment-induced signature	Low	-	-	-	-
	High	0.035^*^	1.515	1.029	2.229
Location	Larynx	-	-	-	-
	Oral cavity	0.352	1.253	0.779	2.014
	Oropharynx	0.014^*^	2.548	1.211	5.359
	Hypopharynx	0.054	3.291	0.979	11.065
Clinical stage	I-III	-	-	-	-
	IVA-C	0.203	1.296	0.869	1.933

CI: Confidence interval; *P* values < 0.05 are shown in bold with asterisks.

**Table 2:** Multivariate survival analysis of recurrence-free survival using Cox proportional-hazard regression in patients with or without platinum-based chemotherapy

**Factor**		***P* value**	**Hazard ratio**	**Lower 95%CI**	**Upper 95%CI**
**Platinum-based chemotherapy received (*n* = 42)**					
*FANCD2*	Low	-	-	-	-
	High	0.011^*^	3.997	1.377	11.599
Location	Larynx	-	-	-	-
	Oral cavity	0.059	3.047	0.958	9.689
	Oropharynx	0.112	2.821	0.786	10.126
	Hypopharynx	0.008	32.262	2.472	421.056
Clinical stage	I-III	-	-	-	-
	IVA-C	0.602	0.766	0.282	2.084
**Without platinum-based chemotherapy (*n* = 250)**					
*FANCD2*	Low	-	-	-	-
	High	0.165	1.375	0.877	2.158
Location	Larynx	-	-	-	-
	Oral cavity	0.310	1.334	0.765	2.324
	Oropharynx	0.065	2.554	0.944	6.910
	Hypopharynx	0.108	3.388	0.766	14.991
Clinical stage	I-III	-	-	-	-
	IVA-C	0.493	1.173	0.744	1.849

CI: Confidence interval; *P* values < 0.05 are indicated in bold with asterisks.

## DISCUSSION

To the best of our knowledge, this is the first study to evaluate the relative contribution of reduced drug uptake and altered molecular responses to the cisplatin resistance phenotype in HNSCC cell lines. This was achieved by comparing concentration-response relationships not only for nominal exposure concentrations but also for resulting intracellular platinum concentrations. The *in vitro* data revealed several important findings. By comparing these two approaches, we estimated that impaired drug uptake accounts for approximately 30% of the observed cisplatin resistance in the two cell lines. The observation of reduced cisplatin uptake in HNO97 cells is supported by our previous data, which showed that HNO97 cells exhibit low levels of the cisplatin uptake transporter *SCL31A1* (human copper transporter 1) and relatively high levels of the cisplatin efflux transporters *ATP7A* and *ATP7B*^[18]^.

Beyond reduced uptake, the remaining ~70% of cisplatin resistance appears to arise from molecular mechanisms, meaning that HNO97 cells respond differently to the drug even when intracellular platinum levels are comparable to those in the sensitive HNO41 cells. Indeed, when both cell lines were exposed to similar intracellular platinum levels (725 pg Pt/µg protein), caspase 3/7 activity in HNO97 cells was delayed in onset and peak compared with HNO41. At the protein level, HNO97 showed no cleavage of caspase 3 or PARP 4 h after the end of the 2.5-h treatment phase, while western blots of HNO41 cells showed strong bands of cleaved caspase 3 and cleaved PARP. At the transcriptional level, several genes involved in cell cycle arrest and DNA damage response were differently regulated. For instance, *GADD45A*, *GADD45G*, and *PPP1R15A* were significantly more strongly induced in HNO41 than in HNO97. Most notably, *CDKN1A* expression increased 14-fold in HNO41 cells but only 3-fold in HNO97 cells. However, under untreated conditions, HNO97 cells already exhibited a 7-fold higher baseline expression of this cell cycle regulator, whose elevated expression has been associated with cisplatin resistance in bladder cancer^[22]^, testicular cancer^[23,24]^, and non-small cell lung cancer^[25]^. Moreover, HNO97 cells exhibited 4- to 8-fold higher baseline mRNA expression levels of *FANCG*, *FANCD2*, *H2AFX*, and other genes involved in DNA damage response and cell cycle arrest compared with HNO41, further substantiating the molecular basis of their cisplatin resistance. This cell line has also been reported to be markedly hypomethylated in the promoter regions of DNA damage response genes, resulting in high expression of *NEIL1*, a bifunctional DNA glycosylase involved in base excision repair^[26]^. Together, these findings strongly suggest that the enhanced DNA damage response capacity of HNO97 cells underlies their cisplatin resistance phenotype. To assess the clinical relevance of the identified genes in HNSCC prognosis, we performed a TCGA-based RNA-seq data set analysis. The results confirmed that the selected genes significantly correlate with recurrence. For instance, high *FANCD2* expression was associated with poor recurrence-free survival in HNSCC in both univariate and multivariate analyses. The specific importance of *FANCD2* was highlighted when the analysis was restricted to patients who received platinum-based chemotherapy. The role of *FANCD2* in cisplatin resistance is further supported by numerous previous studies^[27-30]^, including experimental evidence showing that reducing *FANCD2* expression (e.g., by RNA interference) or inhibiting its protein activity with small molecules sensitizes cancer cells to cisplatin^[31,32]^. In summary, integrating our findings with previously published data, we conclude that altered DNA damage response plays a major role in driving the cisplatin resistance phenotype in HNSCC.

This study has several limitations. First, the HNSCC cell lines were exposed to cisplatin for only 2.5 h, and therefore, the effects of other exposure times could not be evaluated. However, a longer exposure would likely have caused substantial cytotoxic cell loss during treatment. Detached cells would have carried significant amounts of platinum with them, thereby confounding the evaluation of the relationship between intracellular platinum content and anti-proliferative effects, and biasing subsequent analyses through selective enrichment of resistant cells. By contrast, 2.5 h was sufficient to ensure substantial drug uptake while avoiding cellular damage and selection effects during treatment. After 2.5 h of drug exposure, the cells were washed and transferred to drug-free medium. Consequently, all subsequent cellular or molecular effects can only result from the platinum taken up during this period. The plasma half-life of cisplatin is about 30 min^[33]^. After five half-lives, cisplatin should be cleared from plasma and the extracellular space of tumor cells by 96.875%. Thus, an *in vitro* exposure of 2.5 h (mimicking five half-lives) approximates this clinical scenario reasonably well. Second, the cisplatin concentrations used during the 2.5-h exposure for transcriptomic analysis (HNO41: 196 µM; HNO97: 440 µM) were relatively high. However, these supra-pharmacological concentrations were deliberately chosen. They were designed not to kill the cells during exposure but to enable quantifiable intracellular accumulation while imposing sufficient cellular stress, as reflected in gene expression alterations. Overall, the aim was to characterize the downstream responses of two unrelated cell lines when challenged with comparably high intracellular cisplatin levels. Third, anti-proliferative cisplatin effects were normalized to total intracellular platinum rather than to DNA-bound platinum, although previous comprehensive analysis had shown that DNA adduct levels are the most cytotoxic determinant across 19 different HNSCC cell lines. Martens-de Kemp *et al.* reported that cisplatin sensitivity (IC_50_ values ranging from 0.15 to 4 µM) was not correlated with the expression of cisplatin transporters (e.g., *ATP7B*, *SLC31A1*) or DNA damage repair genes (e.g., *ERCC1*), but was highly linked to the amount of platinum bound to DNA (0.1 pmol Pt/µg DNA to > 1 pmol Pt/µg DNA)^[34]^. Measuring DNA adduct levels in our experiments would therefore have provided additional insight. However, increasing evidence indicates that cisplatin targets not only DNA but also RNA, proteins, and lipids^[35-38]^, often resulting in only a weak correlation between DNA platination and cytotoxic efficacy^[39]^. This supports the rationale for quantifying total cellular platinum instead. Fourth, only two representative but unrelated HNSCC cell lines were examined from our panel^[18]^. Although baseline expression of many DNA damage repair genes was higher in the resistant HNO97 line compared to the sensitive HNO41 line, it remains unclear whether this difference is causally linked to resistance. Additional factors - such as apoptotic regulators or oxidative stress response genes not included in the PCR array - may also contribute. This limitation restricts the generalizability of our findings to the broader HNSCC population.

Despite all these weaknesses, it also presents several clear strengths. One key advantage is the quantitative assessment of the relative contribution of drug uptake to the overall resistance phenotype. While other studies have attempted similar analyses using different approaches, their methods had limitations. For instance, in one study of ovarian cancer cell lines (A2780 and a 13-fold more resistant subline), cellular platinum levels after exposure to the respective IC_50_ concentrations (3 and 40 µM) were found to be similar, leading the authors to conclude that drug uptake was the primary factor driving cisplatin resistance^[40]^. In another study, the parental ovarian cancer cell line and several cisplatin-resistant sublines were analyzed for cisplatin IC_50_ values (after 2 h exposure) and drug uptake (also after 2 h). The fold-differences for both IC_50_ values (approximately 3-4-fold higher) and cisplatin accumulation (approximately 3-4-fold lower) in resistant cells closely mirrored each other, leading to a similar conclusion that altered drug uptake was the major contributor to cisplatin resistance in these cell lines^[41]^. However, these conclusions were based on measurements at only a single drug exposure concentration (uptake at IC_50_^[40]^; uptake at 25 µM^[41]^). In contrast, our study evaluated the concentration-response relationship in two complementary ways: first, by plotting nominal drug concentrations in the medium, and second, by measuring the resulting intracellular concentrations. This dual approach provides a more accurate and robust quantification of the impact of altered drug uptake on cisplatin cytotoxicity. A second strength of our study is the detailed analysis of molecular responses at comparable total intracellular platinum concentrations, using a PCR array system that simultaneously quantifies 84 genes involved in DNA damage response and cell cycle arrest. This experimental framework can serve as a template for studies involving other drugs, cell lines, or patient-derived organoids. Finally, the clinical relevance of the identified genes was confirmed through a comprehensive analysis of TCGA data. For instance, *FANCD2* was validated in a retrospective clinical analysis, demonstrating a significant association with poor recurrence-free survival. Importantly, the pharmacological relevance of FANCD2 for cisplatin therapy was highlighted by the observation that its expression impacted survival only in patients receiving platinum-based chemotherapy, and not in the overall HNSCC population.

In conclusion, cisplatin resistance in HNSCC seems to be largely mediated by molecular mechanisms, such as DNA damage repair and cell cycle arrest regulation, and only minimally influenced by reduced cisplatin uptake.

## DECLARATIONS

### Availability of data and materials

All data can be found in the article and its Supplementary Materials. Raw data are available from the corresponding author upon reasonable request.

### Copyright

© The Author(s) 2025.
