# Supplementary material for: Cisplatin resistance in head and neck squamous cell carcinoma is linked to DNA damage response and cell cycle arrest transcriptomics rather than poor drug uptake
Source: Cancer Drug Resist. 2025 Sep 19;8:51. doi: 10.20517/cdr.2025.107 (PMC12539214; doi:10.20517/cdr.2025.107)
Supplement: Supplementary file 1 [file cdr-8-51-SupplementaryMaterials.pdf]

Supplementary Materials

Cisplatin resistance in head and neck squamous cell carcinoma is linked to DNA damage response and cell cycle arrest transcriptomics rather than poor drug uptake

Ketaki Sandu[1,#], Rolf Warta[2,3,#], Uddipta Biswas[4], Wang Zhang[4], Patrick Michl[4], Christel Herold-Mende[2,3], Johanna Weiss[1], Dirk Theile[1]

[1]Internal Medicine IX - Department of Clinical Pharmacology and Pharmacoepidemiology, Heidelberg University, Medical Faculty Heidelberg, Heidelberg University Hospital, Heidelberg 69120, Germany.
[2]Department of Otorhinolaryngology, Head and Neck Surgery, Heidelberg University, Medical Faculty Heidelberg, Heidelberg University Hospital, Heidelberg 69120, Germany.
[3]Division of Experimental Neurosurgery, Department of Neurosurgery, Heidelberg University, Medical Faculty Heidelberg, Heidelberg University Hospital, Heidelberg 69120, Germany.
[4]Internal Medicine IV, Heidelberg University, Medical Faculty Heidelberg, Heidelberg University Hospital, Heidelberg 69120, Germany.
[#]Authors contributed equally.

Correspondence to: Prof. Dirk Theile, Internal Medicine IX - Department of Clinical Pharmacology and Pharmacoepidemiology, Heidelberg University, Medical Faculty Heidelberg, Heidelberg University Hospital, Heidelberg 69120, Germany. E-mail: dirk.theile@med.uni-heidelberg.de

Supplementary Table 1. Changes in gene expression in HNO41 following cisplatin treatment

Gene name	Fold-change	log2 fold-change	P value
GADD45G	25.48	4.67129337248158	0.000387
CDKN1A	13.75	3.78135971352466	0.000000
GADD45A	12.83	3.68144926531496	0.000983
PPP1R15A	8.58	3.10097764772482	0.000337
DDIT3	2.88	1.52606881166759	0.000301
H2AFX	1.85	0.887525270741588	0.001314
XPA	-2.24	-1.163498732	0.00838
REV1	-2.01	-1.007195501	0.00079
SIRT1	1.7	0.765534746362977	0.000499
ABL1	-1.78	-0.831877241	0.008211
ATR	-1.23	-0.298658316	0.003974
CDC25C	-1.27	-0.344828497	0.006044
CRY1	-1.58	-0.659924558	0.001003
RBBP8	-1.58	-0.659924558	0.000598
ATRIP	1.09	0.124328135002202	0.373304
BAX	1.12	0.16349873228288	0.278637
BBC3	2.59	1.37295209791183	0.015325
CDC25A	1.17	0.22650852980868	0.229442
CDK7	1.07	0.0976107966264223	0.31912
CIB1	1.07	0.0976107966264223	0.129169
ERCC1	1.48	0.565597175854225	0.018632
HUS1	1.18	0.238786859587117	0.092654
MBD4	1.04	0.0565835283663675	0.22948
MDC1	1.06	0.0840642647884746	0.614952
OGG1	1.02	0.0285691521967709	0.809733
PNKP	1.05	0.070389327891398	0.544916
PRKDC	1.06	0.0840642647884746	0.126511
RAD18	1.02	0.0285691521967709	0.811874
RAD51B	1.06	0.0840642647884746	0.581671
RAD9A	1.04	0.0565835283663675	0.729851
TP53	1.04	0.0565835283663675	0.647312
APEX1	-1.16	-0.214124805	0.137984
ATM	-1.08	-0.111031312	0.287541
ATRX	-1.07	-0.097610797	0.528759
BARD1	-1.38	-0.464668267	0.053668
BLM	-1.26	-0.333423734	0.087956
BRCA1	-1.15	-0.201633861	0.124097
BRIP1	-1.26	-0.333423734	0.044348
CHEK1	-1.03	-0.042644337	0.813531
CHEK2	-1.34	-0.422233001	0.011496
CSNK2A2	-1.17	-0.22650853	0.145901

DDB1	-1	0	0.955514
DDB2	-1.19	-0.250961574	0.046844
ERCC2	-1.3	-0.378511623	0.179527
EXO1	-1.31	-0.389566812	0.019621
FANCA	-1.04	-0.056583528	0.714128
FANCD2	-1.12	-0.163498732	0.210433
FANCG	-1.17	-0.22650853	0.068444
FEN1	-1.07	-0.097610797	0.159901
LIG1	-1.19	-0.250961574	0.035381
MAPK12	-1.22	-0.286881148	0.083128
MCPH1	-1.33	-0.411426246	0.153643
MLH1	-1.13	-0.176322773	0.129506
MLH3	-1.23	-0.298658316	0.085196
MPG	-1.1	-0.137503524	0.281626
MRE11	-1.21	-0.275007047	0.229615
MSH2	-1.43	-0.516015147	0.02187
MSH3	-1.17	-0.22650853	0.09692
NBN	-1.1	-0.137503524	0.406568
NTHL1	-1.07	-0.097610797	0.139715
PARP1	-1.17	-0.22650853	0.180744
PCNA	-1.14	-0.189033824	0.092047
PMS1	-1.38	-0.464668267	0.045066
PMS2	-1.04	-0.056583528	0.816489
PPM1D	-1.15	-0.201633861	0.183088
RAD1	-1.08	-0.111031312	0.420389
RAD17	-1.4	-0.485426827	0.028869
RAD21	-1.22	-0.286881148	0.114037
RAD50	-1.38	-0.464668267	0.010565
RAD51	-1.38	-0.464668267	0.126268
RNF168	-1.05	-0.070389328	0.68359
RNF8	-1.18	-0.23878686	0.259498
RPA1	-1.17	-0.22650853	0.164612
SMC1A	-1	0	0.996634
SUMO1	-1.23	-0.298658316	0.069092
TOPBP1	-1.26	-0.333423734	0.021749
TP53BP1	-1.1	-0.137503524	0.248714
TP73	-1.34	-0.422233001	0.085635
UNG	-1.11	-0.150559677	0.148541
XPC	-2	-1	0.173518
XRCC1	-1.17	-0.22650853	0.128779
XRCC2	-1.2	-0.263034406	0.151687
XRCC3	-1.18	-0.23878686	0.424499
XRCC6	-1.01	-0.014355293	0.9066

Supplementary Table 2. Changes in gene expression in HNO97 following cisplatin treatment

Gene name	Fold-change	log2 fold-change	P value
GADD45G	16.2	4.0179219079	0.000002
GADD45A	8.29	3.05137210172	0.000181
DDIT3	7.75	2.9541963103	0.000033
PPP1R15A	4.75	2.2479275134	0.000025
CDKN1A	3.08	1.6229303509	0.000001
SIRT1	1.35	0.43295940727	0.003585
REV1	-3.13	-1.646162657	0.000054
TP73	-2.61	-1.384049807	0.000162
MCPH1	-2.48	-1.310340121	0.000038
RBBP8	-2.18	-1.124328135	0.000008
PMS2	-2.15	-1.10433666	0.001146
ABL1	-1.95	-0.963474124	0.000139
APEX1	-1.51	-0.59454855	0.000595
ATRIP	-1.36	-0.443606651	0.003099
ATRX	-1.93	-0.948600847	0.001113
BARD1	-1.61	-0.687060688	0.001161
BAX	-1.28	-0.35614381	0.000526
BLM	-1.88	-0.910732662	0.00113
BRCA1	-1.46	-0.545968369	0.002476
BRIP1	-1.51	-0.59454855	0.004445
CDC25A	-1.51	-0.59454855	0.001208
CDC25C	-1.96	-0.970853654	0.00022
CDK7	-1.28	-0.35614381	0.003006
CHEK1	-1.43	-0.516015147	0.000022
CHEK2	-1.65	-0.722466024	0.005388
CIB1	-1.25	-0.321928095	0.001125
DDB1	-1.62	-0.695993813	0.000528
DDB2	-1.72	-0.782408565	0.00017
ERCC2	-1.74	-0.799087306	0.008196
EXO1	-1.26	-0.333423734	0.004111
FANCA	-1.39	-0.475084883	0.007366
FANCD2	-1.56	-0.641546029	0.000098
MAPK12	-1.64	-0.713695815	0.000587
MDC1	-1.43	-0.516015147	0.000926
MLH1	-1.5	-0.584962501	0.000986
MLH3	-1.72	-0.782408565	0.000536
MPG	-1.35	-0.432959407	0.000437
MRE11	-1.78	-0.831877241	0.002638
MSH2	-1.6	-0.678071905	0.000038
MSH3	-1.7	-0.765534746	0.001327
NTHL1	-1.59	-0.669026766	0.003506

OGG1	-1.43	-0.516015147	0.000945
PARP1	-1.6	-0.678071905	0.000406
PCNA	-1.5	-0.584962501	0.001084
PMS1	-1.45	-0.5360529	0.001634
PPM1D	-1.99	-0.992768431	0.006315
RAD1	-1.36	-0.443606651	0.001086
RAD17	-1.49	-0.575312331	0.001149
RAD21	-1.76	-0.815575429	0.000262
RAD50	-1.52	-0.604071324	0.00074
RAD51	-1.59	-0.669026766	0.000788
RNF168	-1.59	-0.669026766	0.000825
RNF8	-1.84	-0.879705766	0.000577
RPA1	-1.56	-0.641546029	0.000129
SMC1A	-1.59	-0.669026766	0.002027
SUMO1	-1.58	-0.659924558	0.000205
TOPBP1	-1.85	-0.887525271	0.000198
TP53	-1.56	-0.641546029	0.001543
TP53BP1	-1.51	-0.59454855	0.000759
XPC	-1.91	-0.933572638	0.00935
XRCC1	-1.36	-0.443606651	0.000595
XRCC2	-1.97	-0.97819563	0.000153
XRCC3	-1.74	-0.799087306	0.004348
XRCC6	-1.4	-0.485426827	0.002289
BBC3	1.04	0.056583528	0.727172
MBD4	1.01	0.0143552929	0.886589
UNG	1.01	0.0143552929	0.812183
ATM	-1.21	-0.275007047	0.145606
ATR	-1.25	-0.321928095	0.12573
CRY1	-1.03	-0.042644337	0.603647
CSNK2A2	-1.36	-0.443606651	0.010399
ERCC1	-1.19	-0.250961574	0.010539
FANCG	-1.19	-0.250961574	0.037818
FEN1	-1.06	-0.084064265	0.355576
H2AFX	-1.17	-0.22650853	0.052745
HUS1	-1.99	-0.992768431	0.010741
LIG1	-1.39	-0.475084883	0.016336
NBN	-1.33	-0.411426246	0.084762
PNKP	-1.2	-0.263034406	0.010569
PRKDC	-1.22	-0.286881148	0.024583
RAD18	-1.36	-0.443606651	0.012273
RAD51B	-1.56	-0.641546029	0.01366
RAD9A	-1.19	-0.250961574	0.011807
XPA	-1.23	-0.298658316	0.154966

Supplementary Table 3. Baseline differences in gene expression between HNO97 and HNO41

Gene name	Fold-difference	log2 fold-difference	P value
CDKN1A	6.85	2.776103988	0.00008
TP53	6.29	2.653060017	0.000001
OGG1	6.20	2.632268215	0.000031
FANCD2	5.71	2.513490746	0.00001
RAD18	4.79	2.260025656	0.000014
MCPH1	4.39	2.13422094	0.000078
NBN	4.16	2.056583528	0.000042
TOPBP1	4.05	2.017921908	0.000000
RAD50	3.99	1.996388746	0.00002
H2AFX	3.74	1.90303827	0.000001
CDK7	3.41	1.769771739	0.000003
REV1	3.34	1.739848103	0.00002
MBD4	3.3	1.722466024	0.000032
MSH3	3.25	1.700439718	0.000028
XRCC2	3.23	1.691534165	0.000001
FANCG	3.15	1.655351829	0.000007
TP73	3.15	1.655351829	0.000112
MRE11	3.06	1.613531653	0.000081
CDC25C	2.92	1.545968369	0.000000
SIRT1	2.89	1.531069493	0.000012
ERCC2	2.83	1.500802053	0.001291
ATM	2.81	1.49057013	0.000216
CHEK1	2.76	1.464668267	0.000048
DDB2	2.68	1.422233001	0.000115
FANCA	2.65	1.40599236	0.000052
UNG	2.61	1.384049807	0.000191
RAD17	2.6	1.378511623	0.000878
APEX1	2.54	1.344828497	0.000105
HUS1	2.51	1.327687364	0.00249
PCNA	2.48	1.310340121	0.000048
DDB1	2.46	1.298658316	0.000288
BRIP1	2.45	1.292781749	0.000071
MLH1	2.35	1.232660757	0.000142
EXO1	2.34	1.22650853	0.000007
BLM	2.32	1.214124805	0.000059
MSH2	2.32	1.214124805	0.000215
ABL1	2.27	1.182692298	0.000063
SUMO1	2.19	1.13093087	0.000014
RNF8	2.18	1.124328135	0.000001
MPG	2.17	1.117695043	0.000417
TP53BP1	2.17	1.117695043	0.000125

RAD21	2.1	1.070389328	0.000028
BAX	2.09	1.063502942	0.000059
XRCC3	2.09	1.063502942	0.000461
RAD51	2.07	1.049630768	0.001334
PPP1R15A	2.03	1.021479727	0.000292
ATR	1.97	0.97819563	0.000153
PMS1	1.95	0.963474124	0.000687
FEN1	1.93	0.948600847	0.001967
CDC25A	1.82	0.86393845	0.000133
CIB1	1.8	0.847996907	0.001078
RAD1	1.79	0.839959587	0.000776
ATRIP	1.74	0.799087306	0.000154
PPM1D	1.71	0.773996325	0.005501
BRCA1	1.7	0.765534746	0.00002
RPA1	1.67	0.739848103	0.000495
BARD1	1.65	0.722466024	0.001227
CHEK2	1.56	0.641546029	0.001232
RAD9A	1.53	0.613531653	0.001417
CRY1	1.47	0.555816155	0.002395
RNF168	1.47	0.555816155	0.003449
ATRX	1.45	0.5360529	0.001331
BBC3	1.44	0.526068812	0.00793
MDC1	1.37	0.454175893	0.001416
CSNK2A2	1.29	0.367371066	0.006751
LIG1	1.27	0.344828497	0.008745
XRCC1	1.26	0.333423734	0.008077
PMS2	1.24	0.310340121	0.004569
MLH3	1.19	0.250961574	0.00852
MAPK12	-1.21	-0.27500704	0.001713
PRKDC	-1.55	-0.63226821	0.001129
DDIT3	-1.82	-0.86393845	0.00644
RBBP8	-3.08	-1.62293035	0.000067
XPC	1.51	0.59454855	0.290671
SMC1A	1.26	0.333423734	0.017661
XRCC6	1.44	0.526068812	0.040489
RAD51B	1.31	0.389566812	0.020054
XPA	1.3	0.378511623	0.018171
GADD45G	-1.56	-0.64154602	0.022591
PARP1	-1.2	-0.26303440	0.052422
GADD45A	1.06	0.084064265	0.817586
ERCC1	1.02	0.028569152	0.683046
PNKP	-1.02	-0.02856915	0.315587
NTHL1	-1.06	-0.08406426	0.386039
